# Nephroprotective Effect of Aged Black Garlic Extract as a Functional Flock Medicinal on Sodium Benzoate-Induced Chronic Kidney Disease in Albino Rats

**DOI:** 10.3390/life15020217

**Published:** 2025-01-31

**Authors:** Marwa A. Sheir, Ameerah M. Almaski, Manal A. Almughamisi, Suha H. Abduljawad, Essam M. Elsebaie, Rania A. Ahmed

**Affiliations:** 1 Department of Special Food and Nutrition, Food Technology Research Institute, Agricultural Research Center, Giza 3725005, Egypt; 2 Department of Clinical Nutrition, Faculty of Applied Medical Sciences, Taibah University, Universities Road, Medina P.O. Box 344, Saudi Arabiammughamisi@taibahu.edu.sa (M.A.A.); sabduljawad@taibahu.edu.sa (S.H.A.); 3 Food Technology Department, Faculty of Agriculture, Kafrelsheikh University, Kafr El-Sheikh 6860404, Egypt; 4 Department of Zoology, Faculty of Science, Suez University, Suez 43511, Egypt

**Keywords:** caspase-3, IL-1β, nephritis, food preservatives, oxidative stress

## Abstract

Sodium benzoate, a common food preservative, has been linked to oxidative stress, inflammation, and potential damage to various organs, including the kidneys. Aged black garlic (ABG) offers significant potential in supporting body health through its powerful antioxidant and anti-inflammatory properties, which can help reduce cellular damage and inflammation and, thus, improve organ functions. The purpose of this investigation is to investigate the ameliorative effect of aged black garlic extract (ABG extract) on the nephrotoxicity and oxidative stress induced by sodium benzoate. A total of thirty-two adult male albino rats were divided randomly into four groups: Group 1: control; Group 2: orally given ABG extract (200 mg/kg bw) daily for 4 weeks; Group 3: administrated orally by sodium benzoate daily for 4 weeks; Group 4: cotreated with both ABG extract and sodium benzoate for 30 days. This included histological examinations, a histochemical demonstration of DNA contents, and an immunohistochemical demonstration of pro-apoptotic protein caspase-3, as well as a biochemical evaluation of renal MDA, CAT, SOD, GPx, and IL-1β levels. Moreover, serum and urinary urea, uric acid, creatinine, sodium, and potassium levels were also determined, as well as serum C-reactive protein. FI (30 days), FER, and BWG% were calculated as well as urinary volume and protein being measured. The findings revealed that ABG extract significantly improved all histopathological and physiological changes (*p* < 0.05) induced by SB as renal tissue was significantly improved, DNA contents were restored, and capase-3 immunoreactivity was diminished. Additionally, oxidative and inflammatory markers, and renal function parameters, were significantly improved. These results showed that ABG extract possesses significant ameliorative effects against the nephrotoxicity induced by sodium benzoate; this may be mediated by its antioxidant activity.

## 1. Introduction

Kidney diseases, which can be classified into chronic and acute forms, are becoming increasingly prevalent worldwide. Chronic kidney disease (CKD) is characterized by the gradual loss of kidney function over time, often linked to conditions like hypertension, diabetes, and obesity. These diseases can severely impair the kidneys’ ability to filter waste and regulate bodily fluids, leading to serious complications, like kidney failure, which may require dialysis or a kidney transplant for survival. The global rise in kidney disease cases is partly attributed to lifestyle factors, particularly dietary habits [1]. Among the contributing factors, dietary exposure to processed foods containing high levels of preservatives has garnered attention. These additives may accumulate within the kidneys, impairing their filtration capacity and exacerbating cellular damage, especially in individuals already at risk due to conditions such as diabetes or hypertension [2]. The rising prevalence of CKD underscores the need to investigate how dietary factors, including preservatives, influence renal health [3].

Preservatives, such as sodium nitrites, sulfites, and butylated hydroxyanisole (BHA), are frequently used in processed meats, canned goods, and other packaged foods. These additives have been found to potentially cause oxidative stress and inflammation, both of which play a major role in kidney damage [1,4]. Sodium benzoate is a widely used preservative found in a variety of food products, cosmetics, and pharmaceuticals, recognized for its ability to inhibit microbial growth. Despite being classified as Generally Recognized as Safe (GRAS) by regulatory agencies, concerns regarding its potential toxicity have surfaced in recent years, particularly regarding its impact on renal function and overall organ health. For instance, sodium benzoate has been implicated in causing oxidative damage to renal tissues by promoting lipid peroxidation and reducing the activity of key antioxidant enzymes. Understanding these pathways is critical for developing strategies to mitigate the harmful effects of preservatives, particularly in populations at higher risk [5].

Aged black garlic (ABG) is derived from fresh garlic (*Allium sativum*) that undergoes controlled aging under high humidity (80–90%) and moderate heat (typically 60–90 °C) over several weeks. During this process, the garlic’s chemical composition is transformed, giving it a darker color and a softer, sweet-tasting texture. The aging process not only reduces the pungent odor but also enhances the concentration of bioactive compounds with potent antioxidant properties, including S-allyl cysteine (SAC), flavonoids, and polyphenols. These compounds are shown to have therapeutic potential, particularly in anti-inflammatory and antioxidant activities [6]. In addition to its antioxidant properties, ABG demonstrates significant anti-inflammatory effects [7]. Evidence from recent studies highlights that ABG may support cardiovascular health by lowering blood cholesterol and improving lipid profiles [8]. Emerging research has shown that ABG may possess neuroprotective, hepatoprotective, and reproductive property effects due to its high antioxidant and anti-inflammatory properties [9,10,11]. The current work aims to investigate the possible ameliorative impacts of ABG extract against the nephrotoxicity induced by sodium benzoate via histological, histochemical, immunohistochemical, and biochemical analyses.

## 2. Materials and Methods

### 2.1. Materials

SB and other chemicals were purchased from Sigma-Aldrich (St. Louis, MI, USA). Aged black garlic (ABG) is a garlic-processed product that was obtained as 1 gm organic capsules from Mercola Health Resources, LLC, Hoffman Estates, IL, USA. Every chemical employed in this research was of HPLC quality, with 99.9% purity.

One gram of ABG was manually crushed and the crusher was rinsed with ten milliliters of water to collect the extract in a centrifuge tube. The tube was shaken ten times before being left at room temperature for fifteen minutes in an Elma (Singen, Germany) Trans-sonic T460 ultrasonic bath. This was followed by two centrifugations of the mixture for 10 min each at 4000 rpm [12]. After filtering, the supernatant was kept as ABG extract for later examination.

### 2.2. Analysis of the ABG Extract

Total phenolic content was measured using the procedure explained by Singleton and Rossi [13] and the obtained results were explicated as mg gallic acid equivalent (GAE)/g extract. Total flavonoid content (TFC) was measured as explained by Chang et al. [14] and expressed as mg quercetin equivalent (QE)/g extract. Antioxidant activity was measured via the method of ABTS^+^ stated by Re et al. [15] and results were explicated as a µM TEAC (Trolox Equivalent Antioxidant Capacity), i.e., the number of µmoles of Trolox per 1 g.

### 2.3. High-Performance Liquid Chromatography (HPLC) Analysis

The ABG extract was analyzed via Shimadzu LC-10A HPLC instruments (Kyoto, Japan) in a food chemistry laboratory at the National Research Center, Egypt. The phenolic and flavonoid component identifications followed the protocol described by Elsebaie and Essa [16].

### 2.4. Animals and Experimental Design

Thirty-two adult male *Sprague Dawley* rats weighing 170 ± 10 g were included in the present investigation. Rats were housed in plastic cages (8 rats/cage) under standardized environmental conditions at normal atmospheric temperature (25 ± 2 °C) and a normal 12-h light/dark cycle. A rodent diet and water were provided ad libitum throughout the whole experimental period (30 days). For acclimatization and to exclude any infections, rats were kept under observation for one week before experimentation. The entire experimental protocol was carried out according to the Scientific Research Ethics Committee, Egypt (Approval Number: IRB:18-12-2024(7) 18 December 2024). Animals were divided into 4 groups (n = 8):

Group 1: Animals of this group were kept as controls;

Group 2: Animals orally received ABG extract at a dose level of (200 mg/kg bw/daily orally for 30 days) [9];

Group 3: Animals were orally administrated with sodium benzoate (SB) dissolved in distilled water at a dose level of (200 mg/kg bw/daily for 30 days) [17];

Group 4: Animals were treated with both ABG extract (200 mg/kg bw/daily orally for 30 days) and SB (200 mg/kg bw/daily for 30 days).

### 2.5. Histopathological Examinations

At the end of the experiment, animals were dissected and their kidneys were removed and then fixed in 10% neutral formalin, dehydrated, cleared, and embedded in paraffin wax. Paraffin sections of 5 microns in thickness were prepared:

- For histological examination, paraffin sections of five microns of thickness were prepared and stained with a routine hematoxylin and eosin procedure [18];
- For the histochemical demonstration of DNA, the Feulgen reaction was applied [18];
- For the immunohistochemical demonstration of the pre-apoptotic protein (caspase-3) and for immunohistochemical observations, kidney paraffin sections of 4 μm thickness were processed routinely by using a standard avidin-biotin-peroxidase complex system according to the manufacturer’s instructions for pre-apoptotic protein (caspase-3) [19].

### 2.6. Biochemical Studies

#### 2.6.1. Serum and Urine Sampling and Biochemical Analyses

Sera were obtained by centrifugation of the blood samples at 3000 rpm for 15 min and then stored at 20 °C until urea [20], creatinine [21], and uric acid [22] were investigated. For estimation of the C-reactive protein (CRP), sera were obtained by centrifugation of the clotted blood samples, then, a rapid latex agglutination test was applied using a commercial kit obtained from BioDiagnostics, Giza, Egypt, as per the manufacturer’s instructions.

Concerning urine, at the end of the study period, rats in each group were individually housed in metabolic cages for 24 h of urine collection. Total urine volume was measured; then, one ml was collected and used for measurement of the 24 h urinary urea [20], creatinine and protein [21], and uric acid [22]. Sodium and potassium were measured in serum and urine [21]. All serum and urine biochemical investigations were carried out according to the colorimetric method using a spectrophotometer.

#### 2.6.2. Renal Tissue Biochemical Analysis for Antioxidant Markers

For antioxidant demonstration in renal tissue, colorimetric methods via a spectrophotometer were applied. Kidneys were homogenized and centrifuged at 3000 for 20 min; clear supernatants were separated for analyzing malondialdehyde (MDA) [23], superoxide dismutase (SOD) [24], catalase (CAT) [25], and glutathione peroxidase (GPx) [26]. Meanwhile, a renal assay for antiinflammatory Interleukin IL-1β was performed using their respective ELISA kits supplied by Abcam (Waltham, MA, USA).

### 2.7. Biological Evaluations

Every day, the quantities of diet, which were consumed and/or wasted, were recorded while total feed intake (FI) was calculated. Body weight gain % (BWG %) and feed efficiency ratio (FER) were calculated according to Chapman et al. [27] using the following formulas:

(1) $$Body\;Weight\;Gain\;(BWG)\%=\frac{Final\;weight\;\left(g\right)-Intial\;weight\;(g)}{Intial\;weight\;(g)}\times 100$$

(2) $$Feed\;Efficiency\;Ratio\;(FER)=\frac{BodyWeightGain\;(g)}{Feed\;intake\;(g)}$$

### 2.8. Statistical Analysis

Statistical analysis was performed using SPSS v.17 (SPSS Inc., Chicago, IL, USA). Results were articulated as mean ± standard deviation (SD) and all statistical comparisons were made by means of a one-way ANOVA test. A *p* value < 0.05 was considered significant.

## 3. Results

### 3.1. Chemical Properties of ABG Extract

According to data presented in Table 1, it can be noticed that ABG extract contained a high total flavonoid content (56.48 mg QE/100 g) compared with its content of total polyphenols (13.62 mg GAE/g). Also, the results revealed that ABG extract has high antioxidant activity (573.12 µmol TEAC/g). These results were in accordance with those stated by Libero et al. [12].

### 3.2. HPLC Analysis of ABG Extract

The HPLC procedure was used for the fractionation and identification of the polyphenolic and flavonoid components of ABG extract and the results are listed in Table 2.

The data obtained revealed that ABG extract has five phenolic components. P-coumaric acid was the most common compound of the polyphenolic compounds found in the ABG extract (224.1 ppm), followed by chlorogenic acid (156.4 ppm), then gallic acid (109.6 ppm), and caffeic acid (70.2 ppm), with ferulic acid (7.3 ppm) being was the lowest one.

Also, the data in the same table revealed that ABG extract has six flavonoid components. Quercetin was the most common compound of the flavonoid compounds found in the ABG extract (271.4 ppm), followed by catechein (106.5 ppm) and then kaempferol (81.2 ppm), with myricetin (39.8 ppm), epicatechin (35.6 ppm), and gallate epigallocatechin (46.0 ppm) being the lowest ones. These results are in the same line as those obtained by Kim et al. [28].

### 3.3. Histopathological Examination of Kidneys

**Figure 1:**
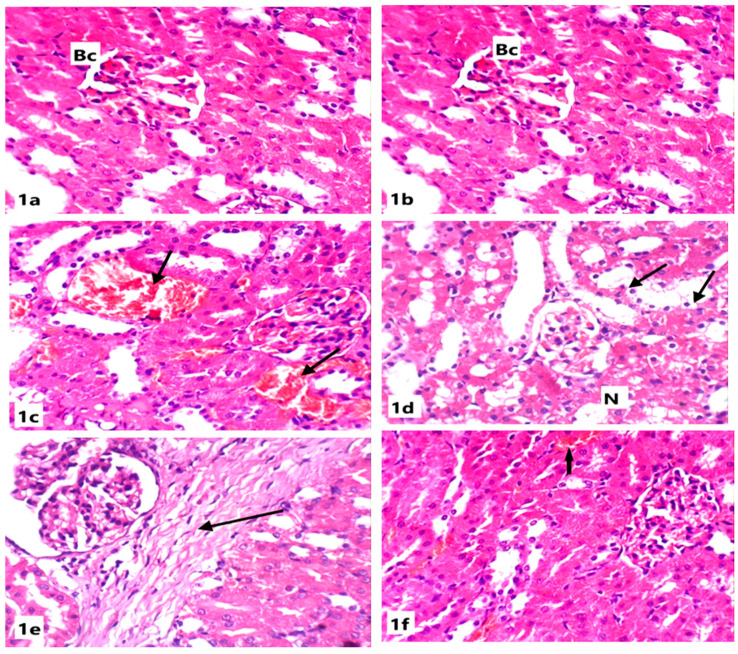
Light microscope photomicrographs of kidney sections of different experimental groups (H&E stain, X400): (**a**): Control group showing normal renal tissue architecture with normal Bowman’s capsule (Bc), glomeruli, and normal tubules. (**b**): Section in the kidney of a rat treated with ABG extract, showing normal renal structure with normal capsules and tubules. (**c**): Section in the kidney of a rat treated with SB showing congested vessels and tubules with internal hemorrhage (arrow). (**d**): Section in the kidney of another rat treated with SB showing necrosis (N) and tubular cytoplasmic vacuolation (arrow). (**e**): Section in the kidney of a rat treated with SB showing intestinal fibrosis (arrow). (**f**): Section in the kidney of a rat treated with ABG extract and SB showing marked improvement of renal tissue with slight hemorrhage (arrow).

Microscopically, the kidneys of rats from the control group revealed the normal renal parenchymal architecture. The kidney is divided into the cortex, containing the renal corpuscles and convoluted tubules, and the medulla, which includes the loops of henle and collecting ducts. In the cortex, the glomeruli appeared as normal spherical or slightly oval structures surrounded by Bowman’s capsules. The proximal tubules have a brush border, enhancing their reabsorption capacity (Figure 1).

Renal tissues of rats from the ABG-extract-treated group did not show any histopathological alterations when compared with control rats as the renal corpuscles and tubules appeared normal (Figure 1a,b). Examination of the renal cortex of SB-treated rats showed congested blood vessels and severe interstitial hemorrhage (Figure 1c,d); tubular degeneration, as some tubules showed complete loss of the lining cells (Figure 1d); atrophy in glomeruli; widening in the Bowman’s capsule; and tubular dilatation (Figure 1e).

Co-treatment with both ABG extract and SB induced a significant improvement of renal tissues in rats from the fourth group when compared with SB-treated group as the renal tissue architecture returned to the normal appearance as corpuscles and tubules appeared more or less like normal and the hemorrhage was markedly declined; however, slight vacuolation of the epithelial lining of some renal tubules and a few intertubular inflammatory cells’ infiltrations were observed in a few sections(Figure 1f).

### 3.4. Histochemical Demonstration of DNA Content in Nuclei

**Figure 2:**
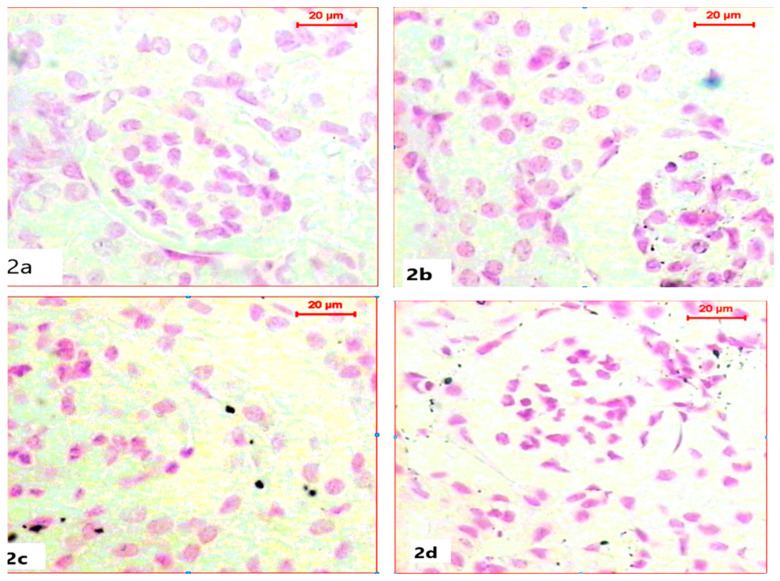
Light microscope photomicrographs of kidney sections of different experimental groups (Fulgen histochemical stain, scale bar 20 µ; DNA in magenta color): (**a**): Control rat showing normal nuclei with normal DNA content. (**b**): Section in the kidney of a rat treated with ABG extract, showing normal renal structure with DNA content. (**c**): Section in the kidney of a rat treated with SB, showing marked reduction in nuclei number with diminished DNA content (**d**): Section in the kidney of a rat treated with ABG extract and SB showing marked improvement of nuclei count with restoration in DNA content.

For the histochemical demonstration of DNA content in nuclei, sections were stained with Feulgen’s reaction to demonstrate DNA as a magenta color. Sections of kidneys of both the control group and ABG-extract-treated groups revealed normal DNA contents in both Bowman capsules and tubules as all nuclei appeared in a spherical shape with a strong magenta color; however, ABG extract did not induce any alteration in DNA content (Figure 2a,b). In a comparison with the control group, SB caused a marked reduction in the number of nuclei and in also in DNA content, which was explained by the mild to moderate magenta color in the renal cells of kidney sections, as well as the decrease in nuclei size (Figure 2c). Renal tissues obtained from rats treated with both ABG extract and SB exhibited marked restoration of nuclei numbers and their strong magenta-colored DNA contents when compared with the SB-treated group (Figure 2d).

### 3.5. Immunohistochemical Demonstration of Pre-Apoptotic Protein (Caspase-3) Activity

**Figure 3:**
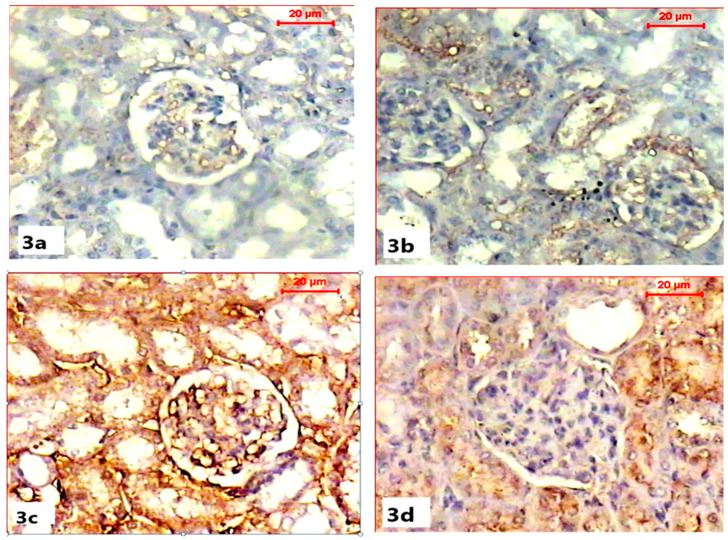
Light microscope photomicrographs showing immunohistochemical alterations among different experimental groups (pre-apoptotic caspase-3 immunohistochemical stain, scale bar 20 µ). (**a**) Section in the kidney of a rat in the control group showing the moderate immune reaction of caspase-3 in cells of the tubules. (**b**) Section in the kidney of rat of ABG-extract-treated group showing moderate expression of caspase-3 immunostain in the renal cells. (**c**) Section in the kidney of a SB-treated rat showing a significant increase in caspase-3 expression (brown color). (**d**) Sections of the kidney of an ABG-extract- and SB-treated rat showing a decrease in caspase-3 expression in renal tubular cells.

Immunohistochemical demonstration of pre-apoptotic protein (caspase-3) activity in the renal tissues of both control and ABG-treated rats revealed the normal absence to mild immunoreactivity of caspase-3 in renal capsules and tubules (Figure 3a,b). Microscopic examination of the renal sections obtained from the SB-treated group showed a marked increase in caspase-3 immunoreactivity with a strong brown color in the renal cortex, especially within Bowman capsules, when compared with the control group (Figure 3c). Co-treatment with ABG extract and SB resulted in the marked inhibition of the caspase-3 reaction as the renal tissue appeared with a mild brown color when compared to SB-treated group (Figure 3d).

### 3.6. Biochemical and Biological Examination

Biochemical investigations in serum, urine, and renal tissue revealed that treating rats with ABG extract only did not induce any pathological alterations as there were not any significant differences between the control group and the ABG-extract-treated group.

**Figure 4:**
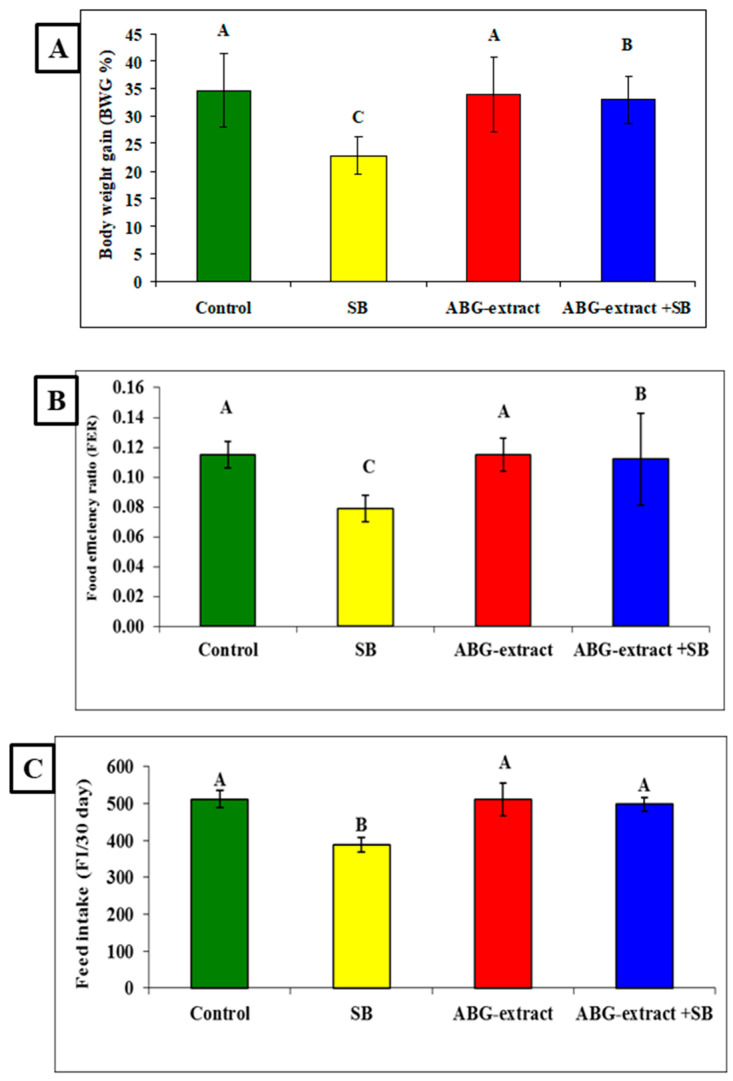
Alterations in (**A**): BWG%, (**B**): FER, and (**C**): FI among experimental groups (mean ± SD, n = 8).

Data in Table 3 and Figure 4 showed significant decreases were recorded in the SB-treated group in the body weight gain (BWG %), food efficiency ratio (FER), and feed intake (FI/30 days) when compared with the control group. Co-treated rats with ABG extract and SB induced significant improvement in the body weight gain (BWG %), food efficiency ratio (FER), and feed intake (FI/30 day) values when compared with the SB-only-treated group.

**Figure 5:**
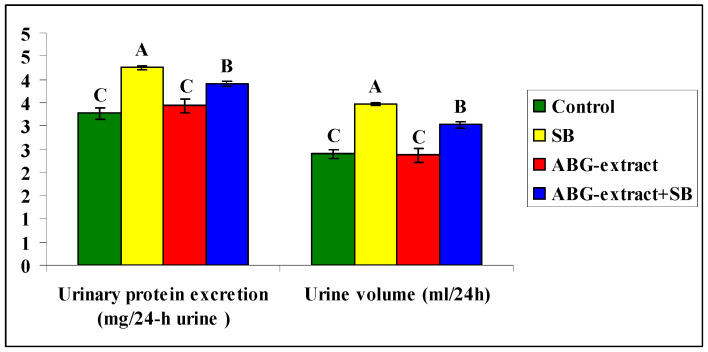
Alterations in Alterations in Urinary protein excretion and urine volume among experimental groups (mean ± SD, n = 8).

Data shown in Table 4 and Figure 5 revealed a significant increase in urinary protein excretion and urine volume caused by SB treatment, by comparing with the control group, which both were diminished as a result of double treatment with ABG extract and SB when compared with the SB-only treated group.

**Figure 6:**
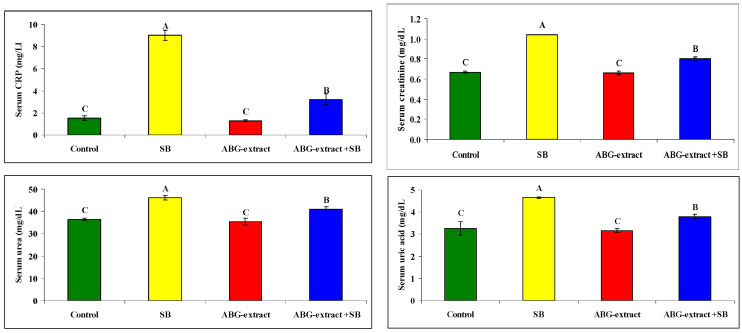

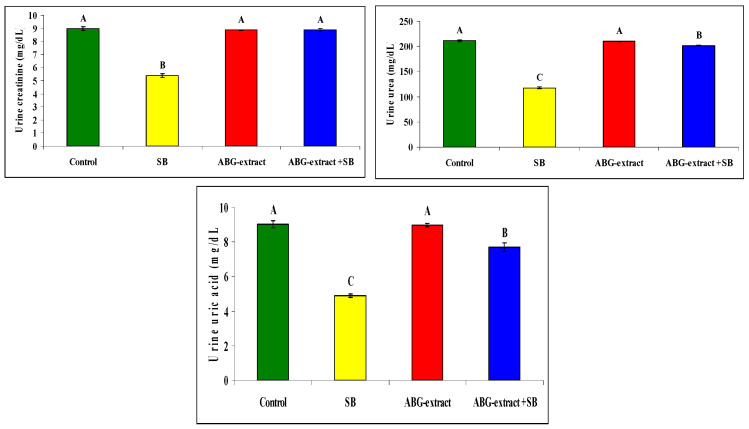
Alterations in CRP, creatinine, urea, and uric acid among experimental groups (mean ± SD, n = 8).

Serum and urine investigations revealed that SB resulted in a significant increase in each of the serum creatinine, serum urea, and serum uric acid, with a significant decrease in urinary creatinine, urinary urea, and urinary uric acid when compared with the control group. On the othesr hand, rats treated with both ABG extract and SB revealed significant decreases in serum urea, uric acid, and creatinine. This was in parallel with the significant increase in urinary urea, uric acid, and creatinine when compared with SB-only-treated group .Concerning the inflammatory status, ABG extract succeeded in modulating renal inflammation as rats treated with both ABG extract and SB revealed a significant improvement in CRP which were elevated significantly in rats treated with SB only, as shown in Table 5 and Figure 6. 

**Figure 7:**
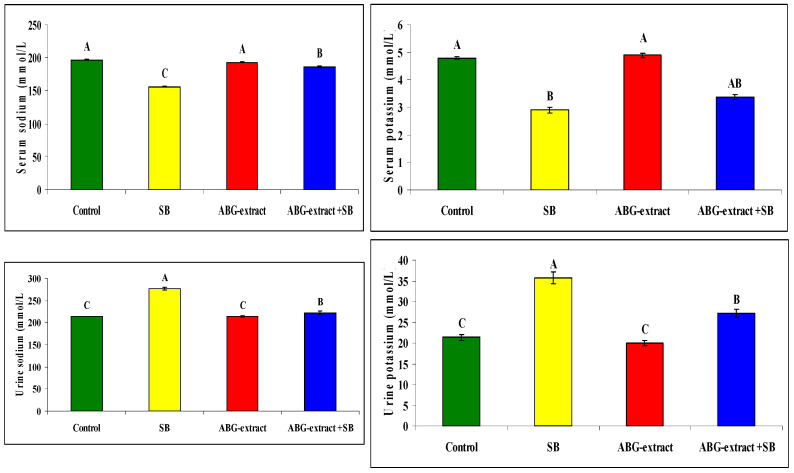
Alterations in sodium and potassium among experimental groups (mean ± SD, n = 8).

Serum sodium and potassium were decreased significantly in SB-treated groups when compared with the control group while they improved significantly and recorded near normal values in the ABG-extract- and SB-treated group when compared with the SB-only-treated group. Meanwhile, urinary sodium and urinary potassium were significantly increased in the SB-treated group when compared with the control group, which was improved by co-treatment by SB and ABG extract as urinary sodium and potassium were significantly increased when compared with SB-treated animals ,as shown in Table 6 and Figure 7

**Figure 8:**
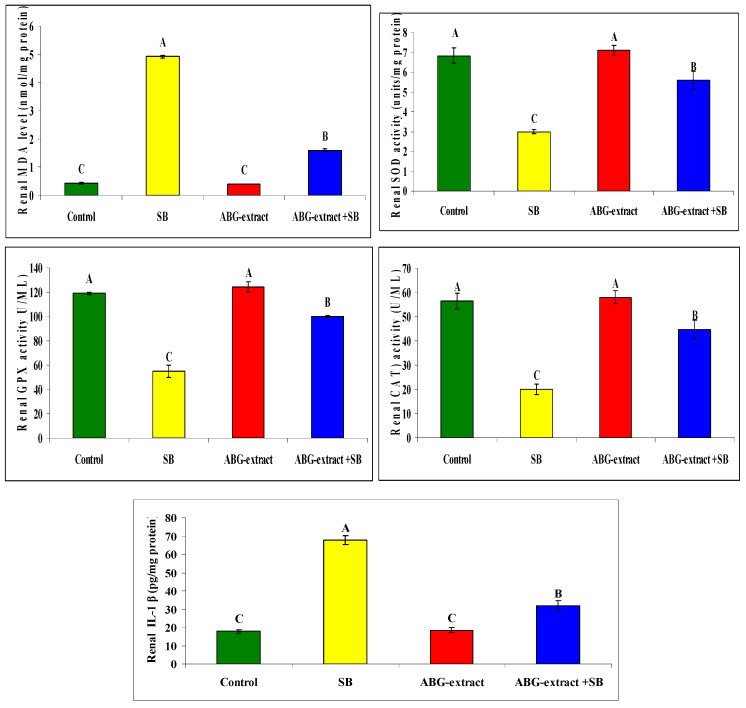
Alterations in renal MDA, SOD, GPx, CAT, IL-1 β among experimental groups (mean ± SD, n = 8).

Regarding oxidative status, treating rats with SB induced a significant increase in renal MDA as well as renal (IL-1 β), and a significant reduction in SOD, GPX, and CAT in comparison with the control group. ABG achieved a marked improvement in oxidative status as rats co-treated with both ABG extract and SB which revealed significant decrease in renal MDA and renal IL-1 β and a significant elevation in renal SOD, GPX, and CAT values in renal tissues in Table 7 and Figure 8.

## 4. Discussion

The safe effect of ABG-extract on body tissues and organs has been discussed in many organs as liver and testis, as the administration of ABG-extract didn’t induce any histopathological or pathophysiological alterations [9,10].

Sodium benzoate (SB) has been proved to induce many pathological alterations in kidney. For instance, a study by Khodaei et al., [29], examined the impact of sodium benzoate in combination with a high-fat diet on the kidneys of mice. The results indicated that sodium benzoate might alter kidney structures, reducing antioxidant defenses and promoting extracellular matrix production, which could be associated with kidney fibrosis. The study highlighted the potential of sodium benzoate to disrupt kidney morphology and suggestively linked it to increased oxidative stress, a common factor in kidney diseases. Moreover, Zeghib et al. [30], studied the effects of sodium benzoate on renal tissues, specifically focusing on the changes in histological markers. They observed that prolonged exposure to sodium benzoate could lead to tissue damage, including inflammation and alterations in kidney architecture, which could contribute to impaired kidney function. In a study on rats, Lee et al. [31] reported that aged black garlic exhibited notable effects on kidney tissues, showing improved cellular structure and reduced pathological changes associated with oxidative stress and acute inflammatory damage. The researchers attributed these benefits to the high antioxidant content in aged black garlic, which helps protect cells from long-term damage. Similarly, Saryono et al. [32] found that aged black garlic mitigated histological changes in fibrotic kidneys in rats, with its anti-inflammatory and antioxidant properties reducing fibrosis and alleviating inflammatory responses. This highlights its potential role as a supportive treatment in chronic kidney injury. Another study by Cheng et al. [33] indicated that aged black garlic extract led to structural improvements in kidney tissue and lowered cellular damage rates in rats exposed to toxic agents. 

The findings suggest that aged black garlic’s components help stabilize cellular health under stress conditions, which could have implications for future therapeutic approaches. A study by Albrakati [34] on rats with induced renal injury found that ABG treatment significantly improved renal histopathology. Kidney sections from ABG-extract treated rats showed reduced glomerular sclerosis, less tubular atrophy, and a decrease in inflammatory cell infiltration compared to control groups. Additionally, there was a notable reduction in the extent of interstitial fibrosis, a key marker of chronic kidney damage. These findings suggest that ABG-extract may help preserve kidney architecture and prevent the progression of renal fibrosis. In another study, You et al. [35] examined the effects of ABG on renal histopathology in rats with chemically induced nephrotoxicity. The results showed that ABG-extract treatment reduced tubular damage, such as tubular dilation and necrosis, and mitigated the accumulation of inflammatory cells. This indicates that ABG-extract has protective effects on renal tubular cells, potentially by enhancing antioxidant defenses and reducing inflammatory pathways. ABG has been investigated for its potential in improving renal histopathology, particularly in models of kidney injury and chronic kidney disease (CKD). Histopathological examination of kidney tissues often reveals structural damage such as glomerular sclerosis, tubular atrophy, interstitial fibrosis, and inflammatory cell infiltration, which are typical markers of renal injury [36]. 

Sodium benzoate (SB), a widely used preservative, has been reported to influence renal markers such as urea, creatinine, and uric acid. These biomarkers are crucial for assessing kidney function and metabolism. In animal studies, Sodium benzoate has been shown to induce kidney toxicity, characterized by elevated levels of creatinine and urea, suggesting renal dysfunction [37]. Sodium benzoate can impact the excretion of nitrogenous waste, which is reflected in altered urea and creatinine levels. A recent study demonstrated that sodium benzoate may affect renal function by modulating ammonia detoxification, which is closely linked to urea synthesis. High doses of SB were associated with increased urea levels, indicating potential renal stress [38]. SB has been shown to influence uric acid levels by altering purine metabolism. It promotes excretion via renal pathways, but excessive intake could disrupt the delicate balance of uric acid synthesis and excretion, potentially leading to hyperuricemia [39]. Additionally, creatinine clearance studies revealed that chronic exposure to sodium benzoate could impair filtration efficiency, particularly in individuals with compromised kidney function [40]. 

The effect of sodium benzoate on serum sodium and potassium levels is not widely studied in isolation, but there is some evidence suggesting that it can indirectly influence electrolyte balance through its impact on kidney function and metabolic processes. Studies have shown that exposure to sodium benzoate may alter kidney function, potentially affecting the regulation of sodium and potassium homeostasis, which are crucial for maintaining proper fluid balance and cellular function [41]. A study by Albrakati [34] demonstrated that ABG supplementation in rats with improved kidney function, as indicated by reduced serum creatinine and blood urea nitrogen (BUN) levels. These results suggest that ABG-extract may help alleviate kidney damage by modulating oxidative stress and improving renal markers. In clinical studies, ABG-extract has been shown to have a protective effect on kidney functions in patients with conditions that predispose them to renal impairment. ABG consumption improved antioxidant capacity and reduced kidney-related markers in patients with diabetes mellitus, a condition that is commonly associated with kidney dysfunction [41].

SB has been shown to induce inflammation in the kidneys, with particular effects on pro-inflammatory cytokines such as C-reactive protein (CRP) and interleukin-1 beta (IL-1β). Elevated CRP levels are typically associated with systemic inflammation, and IL-1β is known for its pivotal role in initiating and amplifying inflammatory responses, especially in kidney tissues. This inflammatory process can contribute to renal damage by promoting the activation of immune cells and the release of additional cytokines, further exacerbating kidney injury [41,42]

Additionally, sodium benzoate has been implicated in the activation of oxidative stress pathways, which, in turn, could stimulate the secretion of CRP and IL-1β, accelerating the inflammatory cascade and leading to more severe renal inflammation. This dual action of sodium benzoate—both as an inducer of oxidative stress and an inflammatory mediator—suggests that prolonged exposure may exacerbate kidney dysfunction [43].

In addition to its antioxidant properties, ABG-extract demonstrates significant anti-inflammatory effects. The fermentation process elevates compounds that suppress pro-inflammatory markers such as interleukin-6 (IL-6) and tumor necrosis factor-alpha (TNF-α). Studies reveal that ABG can inhibit the activation of inflammatory pathways, which is beneficial in managing chronic inflammatory diseases. For instance, in an experimental model, ABG supplementation reduced inflammatory markers in subjects with rheumatoid arthritis, suggesting potential therapeutic applications in managing inflammation-related diseases [31].

Studies have shown that ABG has anti-inflammatory effects, which could further support kidney health. A study by You et al. [35] highlighted that ABG was able to suppress the expression of inflammatory cytokines like TNF-α and IL-6 in animal models, which are often elevated in conditions of renal injury. This reduction in inflammation is significant since chronic inflammation plays a pivotal role in the progression of CKD.

ABG-extract has shown potential in mitigating renal inflammation, a key factor in the development and progression of chronic kidney diseases (CKD). Inflammatory processes in the kidneys are typically characterized by the activation of immune cells and the production of pro-inflammatory cytokines, which can lead to tissue damage and fibrosis. Oxidative stress, which is common in CKD, often exacerbates these inflammatory responses [36]. ABG-extract contains bioactive compounds like S-allyl cysteine (SAC) and other sulfur-containing compounds, which have been found to possess significant anti-inflammatory and antioxidant properties. These compounds may help modulate inflammatory pathways in the kidneys and provide protection against renal injury. Studies on animal models have demonstrated that ABG supplementation can reduce markers of renal inflammation, such as pro-inflammatory cytokines (TNF-α, IL-6, IL-1β), and mitigate kidney damage caused by inflammatory responses [6]. For instance, a study by You et al. [35] investigated the effects of ABG on kidney inflammation in rats with induced renal injury. The results showed that ABG-extract administration led to a reduction in serum levels of inflammatory markers and a decrease in the histological signs of kidney inflammation, such as tubular injury and interstitial fibrosis. This suggests that ABG-extract may play a role in reducing the inflammatory cascade in the kidneys. Similarly, a study by Lee et al. [42] demonstrated that ABG suppressed the expression of nuclear factor-kappa B (NF-κB), a key regulator of inflammation. By inhibiting NF-κB activation, ABG-extract could prevent the transcription of inflammatory cytokines and reduce kidney inflammation. This anti-inflammatory effect may be beneficial in conditions such as diabetic nephropathy or hypertensive nephropathy, where renal inflammation plays a major role in disease progression. Khan et al. [44] demonstrated that sodium benzoate-induced oxidative stress disrupts cellular homeostasis, activating pro-apoptotic proteins such as Bax and reducing the levels of anti-apoptotic proteins like Bcl-2. This imbalance further drives kidney cell death, contributing to the pathogenesis of renal diseases. The study emphasized the role of sodium benzoate in initiating both oxidative damage and apoptotic cell death in renal tissues. Additionally, sodium benzoate can interact with cellular structures, promoting apoptosis (cell death) and potentially contributing to long-term damage to kidney cells [45].

Wali et al. [46] examined the molecular mechanisms underlying sodium benzoate-induced kidney damage. Their study found that sodium benzoate exposure increased oxidative stress markers and activated caspase-3, a key enzyme involved in apoptosis. The activation of caspase-3 leads to cellular disintegration and further kidney dysfunction. This study highlights the direct connection between oxidative damage and cell death, further exacerbating kidney impairment and fibrosis.

In another investigation, Akter et al. [47] discussed the impact of environmental toxins, including sodium benzoate, on renal health. They reported that prolonged exposure to sodium benzoate leads to increased ROS generation, resulting in inflammation and apoptosis within kidney cells. These findings are consistent with previous research showing that ROS can trigger various signaling pathways, including the JNK and p38 MAPK pathways, which are known to mediate apoptotic cell death and inflammation.

One of the key concerns surrounding sodium benzoate is its ability to induce oxidative stress and inflammation, which can lead to kidney dysfunction. Studies have indicated that sodium benzoate can increase the production of free radicals, leading to cellular damage within renal tissues. The oxidative damage is thought to be a significant contributing factor to nephrotoxicity, as it disrupts the normal functioning of kidney cells and tissues. In animal studies, prolonged exposure to sodium benzoate has shown an increase in markers of kidney injury, such as serum creatinine and urea levels, signaling renal impairment [48]. Further research has also highlighted the relationship between sodium benzoate and changes in metabolic profiles, including glucose metabolism. Sodium benzoate’s metabolism in the liver and kidneys produces hippurate, which can accumulate over time. Some studies have shown that elevated levels of hippurate may exacerbate renal damage, particularly in individuals with pre-existing kidney conditions [45]. Moreover, it has been suggested that chronic exposure to sodium benzoate could lead to hyperglycemia, which places additional strain on the kidneys and could promote the development of renal diseases such as diabetic nephropathy [46]. 

According to Khan et al. [44], sodium benzoate induces oxidative stress in renal tissues, leading to the activation of apoptotic pathways. This was demonstrated by increased levels of oxidative markers such as malondialdehyde (MDA) and decreased antioxidant enzyme activities like superoxide dismutase (SOD) and catalase (CAT). The accumulation of ROS in kidney cells is believed to disrupt mitochondrial function, leading to further cell damage and triggering apoptosis through the mitochondrial pathway.

The cumulative evidence from these studies suggests that sodium benzoate’s ability to generate ROS and activate apoptotic pathways plays a crucial role in kidney damage. This underscores the importance of regulating sodium benzoate consumption and further investigating its long-term effects on renal health. Exposure to SB has been linked to oxidative stress in kidney tissues through various mechanisms. Sodium benzoate increases reactive oxygen species (ROS) production, leading to cellular damage. This is particularly evident in studies on animal models, which show elevated levels of malondialdehyde (MDA), a marker for lipid peroxidation, alongside decreased antioxidant enzyme activities such as superoxide dismutase (SOD) and catalase in the kidneys. These findings suggest that sodium benzoate impairs the balance between pro-oxidants and antioxidants, promoting oxidative stress and subsequent renal injury [38].

Furthermore, oxidative stress induced by sodium benzoate is closely associated with mitochondrial dysfunction. Mitochondrial pathways play a critical role in ROS production, and sodium benzoate has been shown to disrupt mitochondrial integrity, leading to apoptosis and necrosis in kidney cells. Such damage can exacerbate chronic kidney diseases when exposure is prolonged .In addition to ROS production, sodium benzoate-induced mitochondrial dysfunction has been highlighted as a key contributor to renal oxidative stress. Mitochondria, being central to cellular energy metabolism, are particularly vulnerable to oxidative insults. Damage to mitochondrial membranes and DNA can result in apoptosis and necrosis of kidney cells, aggravating chronic renal diseases under prolonged exposure to SB [43]. One study by Khan et al. [44] found that high levels of sodium benzoate could result in oxidative stress, which may, in turn, lead to disturbances in electrolyte levels, including sodium and potassium. This disturbance is primarily related to the renal filtration process, where the kidneys help maintain electrolyte balance. It has been suggested that prolonged exposure to sodium benzoate could impair kidney function, indirectly affecting the serum concentrations of key electrolytes more research is needed to specifically assess the direct impact of sodium benzoate on serum sodium and potassium levels, especially in long-term exposure scenarios or at higher concentrations.

On the other hand, aged black garlic has gained attention for its antioxidant properties, which could potentially counteract oxidative damage caused by harmful substances like sodium benzoate. Some studies have shown that aged black garlic might reduce oxidative stress and mitigate inflammation, both of which are associated with kidney injury [49]. Its components, such as S-allyl cysteine, are believed to have protective effects on tissues and organs, possibly extending to the kidneys [50]

ABG-extract has garnered attention for its potential health benefits, particularly in reducing oxidative stress. Oxidative stress is a condition characterized by an imbalance between reactive oxygen species (ROS) and the body’s ability to detoxify them or repair the resulting damage. This imbalance can contribute to a range of chronic diseases, including cardiovascular disease, diabetes, and cancer [51]. 

Aged black garlic, which is produced through a fermentation process that typically lasts for several weeks, has been found to contain higher levels of certain bioactive compounds, such as S-allyl cysteine (SAC), which have antioxidant properties. Studies suggest that ABG can help mitigate oxidative stress by increasing the body’s antioxidant capacity. For instance, it has been shown to upregulate antioxidant enzymes like superoxide dismutase (SOD) and catalase, which play a crucial role in neutralizing ROS [52].

Furthermore, research indicates that ABG may reduce markers of oxidative stress in various animal and human studies. For example, in a study by You et al. [35], ABG supplementation in mice led to a significant reduction in malondialdehyde (MDA) levels, a marker of lipid peroxidation, indicating reduced oxidative damage.

In humans, a clinical trial by Lee et al. [42] demonstrated that regular consumption of ABG improved the antioxidant status in patients with metabolic syndrome, which is often associated with increased oxidative stress. This suggests that ABG may be a valuable dietary supplement in managing oxidative stress-related conditions.

In addition to its direct anti-inflammatory effects, ABG-extract may also reduce oxidative stress, which is often a trigger for renal inflammation. A study by Ahmed & Wang [53] showed that ABG increased the activity of antioxidant enzymes like superoxide dismutase (SOD) and catalase, thereby reducing oxidative damage and, consequently, inflammation in renal tissues

## 5. Conclusions

The findings of this study demonstrated that ABG extract significantly ameliorated the nephrotoxicity induced by sodium benzoate in rats by improving alterations and histological, histochemical, immunohistochemical, biochemical, and antioxidant levels induced by sodium benzoate. The current study recommends aged black garlic as a herbal medication for renal diseases.

## Figures and Tables

**Table 1:** Chemical properties of ABG extract.

Chemical Properties	ABG Extract
Total polyphenol content (mg GAE/g)	13.62
Total flavonoids (mg QE/100 g)	56.48
Antioxidant activity (µmol TEAC/g)	573.12

**Table 2:** Major phenolic and flavonoid compounds (ppm) identified in ABG extract by HPLC.

Components	Concentration (ppm)
**Phenolic compounds**	
Chlorogenic acid	156.4
Caffeic acid	70.2
Gallic acid	109.6
Ferulic acid	7.3
P-coumaric acid	224.1
**Flavonoid compounds**	
Catechin	106.5
Gallate epigallocatechin	46.0
Epicatechin	35.6
Quercetin	271.4
Myricetin	39.8
Kaempferol	81.2

**Table 3:** Alterations in BWG%, FER, and FI among experimental groups (mean ± SD, n = 8).

Parameters	Groups			
	Control	SB	ABG Extract	ABG Extract and SB
Body weight gain (BWG %)	34.70 ± 6.80 ^A^	22.80 ± 3.40 ^C^	33.91 ± 6.71 ^A^	32.90 ± 4.30 ^B^
Food efficiency ratio (FER)	0.115 + 0.009 ^A^	0.079 ± 0.009 ^C^	0.115 ± 0.011 ^A^	0.112 ± 0.031 ^B^
Feed intake (FI/30 day.)	512.15 ± 22.29 ^A^	389.07 ± 19.6 ^B^	510.85 ± 44.80 ^A^	497.52 ± 18.09 ^A^

A, B, and C: There is no significant difference (*p* > 0.05) between any two means, within the same row, that have the same superscript letter.

**Table 4:** Alterations in urinary protein excretion and urine volume among experimental groups (mean ± SD, n = 8).

Parameters	Groups			
	Control	SB	ABG Extract	ABG Extract and SB
Urinary protein excretion (mg/24-h urine)	3.27 ± 0.13 ^C^	4.26 ± 0.04 ^A^	3.43 ± 0.15 ^C^	3.90 ± 0.06 ^B^
Urine volume (ml/24 h)	2.40 ± 0.10 ^C^	3.47 ± 0.04 ^A^	2.37 ± 0.15 ^C^	3.02 ± 0.07 ^B^

A, B, and C: There is no significant difference (*p* > 0.05) between any two means, within the same row, that have the same superscript letter.

**Table 5:** Alterations in CRP, creatinine, urea, and uric acid among the experimental groups (mean ± SD, n = 8).

Parameters	Groups			
	Control	SB	ABG Extract	ABG Extract and SB
Serum Crp (mg/dL)	1.53 ± 0.21 ^C^	9.03 ± 0.45 ^A^	1.30 ± 0.10 ^C^	3.23 ± 0.49 ^B^
Serum creatinine (mg/dL)	0.67 ± 0.01 ^C^	1.04 ± 0.00 ^A^	0.66 ± 0.02 ^C^	0.80 ± 0.02 ^B^
Serum urea (mg/dL)	36.56 ± 0.57 ^C^	46.10 ± 1.01 ^A^	35.34 ± 1.53 ^C^	41.00 ± 1.00 ^B^
Serum uric acid (mg/dL)	3.27 ± 0.31 ^C^	4.66 ± 0.04 ^A^	3.17 ± 0.09 ^C^	3.80 ± 0.10 ^B^
Urine creatinine (mg/dL)	8.98 ± 0.14 ^A^	5.37 ± 0.15 ^B^	8.87 ± 0.03 ^A^	8.90 ± 0.10 ^A^
Urine urea (mg/dL)	211.27 ± 2.37 ^A^	117.5 ± 1.8 ^C^	210.00 ± 1.00 ^A^	202.11 ± 0.84 ^B^
Urine uric acid (mg/dL)	9.03 ± 0.18 ^A^	4.90 ± 0.10 ^C^	9.00 ± 0.10 ^A^	7.71 ± 0.25 ^B^

A, B, and C: There is no significant difference (*p* > 0.05) between any two means, within the same row, that have the same superscript letter.

**Table 6:** Alterations in sodium and potassium among experimental groups (mean ± SD, n = 8).

Parameters	Groups			
	Control	SB	ABG Extract	ABG Extract and SB
Serum sodium (mmol/L)	196.77 ± 0.87 ^A^	156.10 ± 0.85 ^C^	193.00 ± 1.00 ^A^	186.33 ± 1.53 ^B^
Serum potassium (mmol/L)	4.79 ± 0.06 ^A^	2.90 ± 0.10 ^B^	4.89 ± 0.07 ^A^	3.36 ± 2.91 ^AB^
Urine sodium (mmol/L)	214.13 ± 0.42^C^	277.07 ± 2.61 ^A^	213.73 ± 1.42 ^C^	222.33 ± 3.51 ^B^
Urine potassium (mmol/L)	21.37 ± 0.71 ^C^	35.70 ± 1.47 ^A^	19.97 ± 0.55 ^C^	27.13 ± 1.03 ^B^

A, B, and C: There is no significant difference (*p* > 0.05) between any two means, within the same row, that have the same superscript letter.

**Table 7:** Alterations in renal MDA, SOD, GPx, CAT, and IL-1 **β** among experimental groups (mean ± SD, n = 8).

Parameters	Groups			
	Control	SB	ABG Extract	ABG Extract and SB
Renal MDA level (nmol/mg protein)	0.43 ± 0.03 ^C^	4.92 ± 0.05 ^A^	0.40 ± 0.01 ^C^	1.60 ± 0.05 ^B^
Renal SOD activity (units/mg protein)	6.83 ± 0.40 ^A^	3.00 ± 0.10 ^C^	7.10 ± 0.26^A^	5.60 ± 0.46 ^B^
Renal GPX activity (U/ML)	119.00 ± 1.00 ^A^	55.00 ± 5.00 ^C^	124.33 ± 4.04 ^A^	100.00 ± 1.00 ^B^
Renal CAT) activity (U/ML)	56.33 ± 3.21 ^A^	20.00 ± 2.00 ^C^	58.00 ± 2.65 ^A^	44.67 ± 3.79 ^B^
Renal IL-1 β (pg/mg protein)	17.83 ± 1.04 ^C^	67.67 ± 2.52 ^A^	18.5 ± 1.32 ^C^	32.00 ± 2.65 ^B^

A, B, and C: There is no significant difference (*p* > 0.05) between any two means, within the same row, that have the same superscript letter.

## Data Availability

The authors confirm that the data supporting the findings of this study are available within the article.
